# Hematology, Biochemistry, and Protein Electrophoresis Reference Intervals of Western European Hedgehog (*Erinaceus europaeus*) from a Rehabilitation Center in Northern Portugal

**DOI:** 10.3390/ani13061009

**Published:** 2023-03-10

**Authors:** Sofia Rosa, Ana C. Silvestre-Ferreira, Roberto Sargo, Filipe Silva, Felisbina Luísa Queiroga

**Affiliations:** 1 School of Life and Environmental Sciences (ECVA), University of Trás-os-Montes and Alto Douro (UTAD), Quinta dos Prados, 5001-801 Vila Real, Portugal; 2 Department of Veterinary Science, School of Agrarian and Veterinary Sciences (ECAV), University of Trás-os-Montes and Alto Douro (UTAD), Quinta dos Prados, 5001-801 Vila Real, Portugal; 3 Animal and Veterinary Research Centre (CECAV), University of Trás-os-Montes and Alto Douro (UTAD), Quinta dos Prados, 5001-801 Vila Real, Portugal; 4 Associated Laboratory for Animal and Veterinary Science (AL4AnimalS), University of Trás-os-Montes and Alto Douro (UTAD), Quinta dos Prados, 5001-801 Vila Real, Portugal; 5 Veterinary Teaching Hospital, University of Trás-os-Montes and Alto Douro (HVUTAD), Quinta dos Prados, 5001-801 Vila Real, Portugal

**Keywords:** Western European hedgehog, *Erinaceus europaeus*, hematology, biochemistry, protein electrophoresis, reference intervals

## Abstract

**Simple Summary:**

The Western European hedgehog (*Erinaceus europaeus*) is an insectivorous mammal with a wide geographic distribution. Owing mostly to climate changes and anthropogenic pressures, a considerable number of hedgehogs now live in urban areas close to humans, where they are exposed to contaminants and biological agents that may result in disease with the correspondent hematological and biochemical alterations. Hedgehogs can work as bioindicators to environmental pollution and host multiple zoonotic agents, making them relevant for One Health studies. Thus, it is essential to deepen the knowledge on this species and calculate reference intervals for the usual hematological and biochemical parameters. This would make it possible to recognize the “normal” and identify the “disease”. In this study, some significant differences were evident, especially when comparing age groups (juveniles versus adults), showing the relevance of further investigations in this species.

**Abstract:**

The Western European hedgehog (*Erinaceus europaeus)* can work as a bioindicator of environmental pollution and be a host for multiple zoonotic agents, making it relevant in terms of One Health studies. It is essential to deepen the knowledge on this species and calculate reference intervals (RIs) for the usual hematological and biochemical parameters. For this retrospective study (2017–2022), the archives of the Clinical Pathology Laboratory (LPC) of University of Trás-os-Montes and Alto Douro (UTAD) Veterinary Teaching Hospital were analyzed. Data of hematology, clinical biochemistry, and protein electrophoresis from 37 healthy hedgehogs of the Wild Animal Rehabilitation Center at UTAD, Northern Portugal, were included. It was possible to calculate RIs for almost all of the variables in the study, using Reference Value Advisor V2.1. Moreover, sex and age effects were investigated: alkaline phosphatase (*p* = 0.012, higher in males); total proteins (*p* = 0.034, higher in adults); mean cell volume (*p* = 0.007) and mean corpuscular hemoglobin (*p* = 0.010) (both higher in juveniles); and red blood cell distribution width (*p* = 0.021, higher in adults). Our study allowed for the first time to define RIs for a population of hedgehogs in Portugal, having a potentially relevant impact on species conservation and in the human–animal health interface.

## 1. Introduction

The Western European hedgehog, *Erinaceus europaeus*, belongs to the mammal order Eulipotyphla, family Erinaceidae, subfamily Erinaceinae, and genera *Erinaceus* [1]. There are two species of hedgehog in Europe, *Erinaceus europaeus* and *Erinaceus roumanicus*. *E. europaeus* may be found in western and central Europe, including Britain, the Mediterranean Islands, southern Scandinavia, and into Estonia and northern Russia [2,3]. It is also the most common one in Portugal [4,5].

The species *E. europaeus* is on the International Union for the Conservation of Nature (IUCN) and Red Book of Vertebrates of Portugal (LVVP) red list, as being “least concern” because it is common and abundant throughout its wide range [5,6]. However, in the last decades, there are registers of a decrease in the number of individuals of this species [7] which can be justified by several factors: the Western European hedgehog has some natural predators that pose a threat to the survival of the species (badgers (*Meles meles*) are the most significant ones) [8,9]; as the hedgehog occupies agricultural areas, it is frequently exposed to poisoning by pesticides and rodenticides [10]; the transformation and fragmentation of its habitat, as well as climate changes, affect its survival [11]; they are one of the vertebrates that frequently suffer mortality owing to road traffic [7]; and finally, they harbor a wide variety of different parasites and pathogens [8,12,13].

Owing to different pressure factors, hedgehogs are moving close to humans and urban centers, adapting to new habitats, food resources (as pet food), and refugia (as public and private gardens) [11,14,15,16], where they are exposed to contaminants and biological agents, which may result in disease with the correspondent hematological and biochemical alterations [17]. Furthermore, hedgehogs’ ecological and feeding habits, as well as their high population densities and repeated contacts with wild and domestic animals and humans, make this species a possible sentinel for a One Health approach, mainly owing to its possible involvement in the ecology of potentially emerging pathogens [18,19], such as endoparasites (*Crenosoma striatum*, *Capillaria aerophila* (syn. *Eucoleus aerophilus*), *Capillaria* spp., coccidia, *Cryptosporidium* spp., *Brachylaemus* spp., and *Capillaria hepatica)* [12]. Published systematic reviews show that *E. europaeus* may harbor zoonotic pathogens and that the species can play an important role in the epidemiology of various zoonotic infections. The prevalence of zoonotic agents in hedgehogs, from both urban and rural habitats, is of major concern because there is a high probability of contact with humans and companion animals. Recently, several studies have shown that *E. europaeus* can harbor methicillin-resistant *Staphylococcus aureus* (MRSA) and that this resistance can predate the human discovery of antibiotics [20,21]. For this reason, studying local *E. europaeus* populations is highly relevant [22,23].

Reference intervals (RIs) are ranges calculated from a group or population of healthy individuals of a given species and are most widely used as a medical decision-making tool, serving as the basis of laboratory testing, differentiating whether or not a patient is healthy [24]. In order to determine RIs, the American Society of Veterinary Clinical Pathology (ASVCP) established guidelines with specific veterinary recommendations where it is necessary to define the population of interest as well as criteria to confirm the health status of selected individuals [25]. The studies on hematological and biochemical profiles for the species *E. europaeus* are scarce [17,26,27]. The most recent study, performed in a rehabilitation center in Italy (2014), evaluated hematological and biochemical profiles in the species, creating their own reference intervals [17].

In Portugal, as far as we know, there are no records on the subject. Therefore, it was our purpose to carry out a study on the hematological and biochemical (routine and electrophoretic) profiles of *E. europaeus* in a population of healthy individuals from a wildlife rehabilitation center in Northern Portugal, in order to create reference intervals, and thus contributing to species conservation and a better understanding of the impact in the human–hedgehogs health interface.

## 2. Materials and Methods

**Figure 1:**
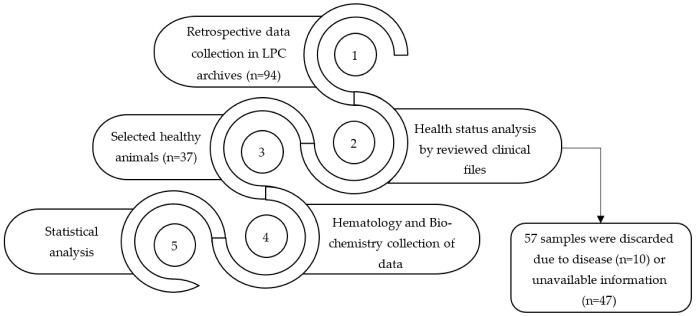
Flowchart of the retrospective analysis of this study.

This retrospective study was carried out at the Clinical Pathology Laboratory of the Veterinary Teaching Hospital of the University of Trás-os-Montes and Alto Douro (LPC-HVUTAD), located in the city of Vila Real, Portugal. A total of 94 registers of *E. europaeus* were identified from the LPC archive, corresponding to the routine post-quarantine evaluation of admitted individuals. The clinical files were reviewed to access the health status of animals on the date of blood collection. Animals that had normal physical examinations, no alterations on the diagnostic exams (whole body X-rays, blood collection, and coprological examination), and no signs of illness until the date of release were considered healthy. By reviewing clinical files, it was possible to identify 37 cases of healthy animals, thus 57 cases were discarded owing to several causes of disease (*n* = 10) or unavailable information (*n* = 47) (Figure 1). All of the hedgehogs included in this study originated from the region of northern Portugal, including the Douro River basin, and were received at the Wild Animal Rehabilitation Center of the Veterinary Teaching Hospital of UTAD (CRAS-HVUTAD) between 1 March 2017 and 31 August 2022. Age was evaluated at admission by a veterinarian, based on morphometrics, after Haigh et al. (2014) [28]. Only independent juveniles and adult animals were admitted to this study, being assigned on the day of blood collection to one of the two categories [28]. Sex was determined visually by the observation of the external genital organs. The entire process of animal capture and sample collection was carried out by veterinarians and qualified auxiliary personnel. Only animals considered healthy were enrolled. For this study, no ethical approvals were required, as all blood samples were routinely collected for official diagnostic and monitoring purposes and subsequently made available to this retrospective study.

### 2.1. Sample Collection

Animals were anesthetized by mask administration of 5% isoflurane in an oxygen flow of 2 L/min. After the loss of reflexes, the isoflurane concentration was diminished to 2% and maintained during the physical exam and sample collection. Blood samples were obtained through a cranial vein cava punction with 25 G needles and 1 mL syringes, always with the animal under anesthesia. Blood was transferred into 0.5 mL lithium heparin tubes (FL Medical). A complete record was kept for each animal, including sex, age group, and date of capture. After collection, all samples were transported to the LPC-HVUTAD for analysis.

### 2.2. Sample Treatment and Processing

For hematology, blood samples were analyzed in the ProCyte Dx (IDEXX) hematology analyzer and the following parameters were determined: red blood cells (M/μL), hematocrit (%), hemoglobin (g/dL), mean cell volume (fL), mean corpuscular hemoglobin (pg), mean corpuscular hemoglobin concentration (g/dL), red blood cell distribution width (%), reticulocyte percent, reticulocyte count (K/μL), white blood cell (K/μL), neutrophil percent, lymphocyte percent, monocyte percent, eosinophil percent, basophil percent, neutrophil count (K/μL), lymphocyte count (K/μL), monocyte count (K/μL), eosinophil count (K/μL), basophil count (K/μL), platelet count (K/μL), mean platelet volume (fL), and plateletcrit (%). There is no predefined menu for hedgehogs in this analyzer; in addition, the option used was “others”, with no associated reference ranges for any parameter.

Plasma samples were obtained through routine centrifugation at the SELECTA Centromix-BLT centrifuge, for 5 min at 2618× *g*. Then, it was transferred into 2 mL aliquots, properly identified, and kept at −20 °C until analysis. According to laboratory standards, strongly hemolyzed or lipemic samples were rejected. For the biochemical analysis, the DiaSys Respons920 Clinical Chemistry Analyzer was used and the following parameters were determined: glucose (mg/dL), total proteins (g/dL), albumin (g/dL), alanine aminotransferase (U/L), alkaline phosphatase (U/L), creatinine (mg/dL), urea (mg/dL), phosphorus (mg/dL), total calcium (mg/dL), cholesterol (mg/dL), triglycerides (mg/dL), gamma-GT (U/L), globulins (g/dL), aspartate aminotransferase (U/L), total bilirubin (mg/dL), sodium (mmol/L), potassium (mmol/L), and chloride (mmol/L).

The remaining plasma was then used for protein electrophoresis on the Elephor8S automatic electrophoresis analyzer, following the manufacturer’s instructions.

### 2.3. Data Collection Methodology, Statistical Analysis, and Reference Intervals

Data were registered in an Excel sheet and RIs were established according to the ASVCP guidelines using the Reference Value Advisor V2.1 program [16].

To investigate the influence of sex and age in the analyzed parameters, a statistical analysis was performed using the SPSS program, version 27. The Shapiro–Wilk test was performed for testing samples’ normality and then samples were subjected to analysis of variance (ANOVA) to investigate the influence of sex and age. A value of *p* < 0.05 was considered significant.

## 3. Results

### 3.1. Descriptive Analysis of the Total Number of Animals

Regarding sex, information was only available for 35 of the 37 animals: 13 (35.14%) males and 22 (59.46%) females; information was missing in 2 cases (5.41%).

For age, information was available in 34 cases of the 37 cases: 20 (54.05%) juveniles and 14 (37.84%) adults; it was not possible to obtain the respective information for the remaining 3 (8.11%) samples.

### 3.2. Reference Intervals

For hematology, Table 1 describes the RIs of the evaluated parameters in the total sample (*n* = 37).

For routine plasma biochemistry and protein electrophoresis, the results are presented in Table 2 (*n* = 37).

**Figure 2:**
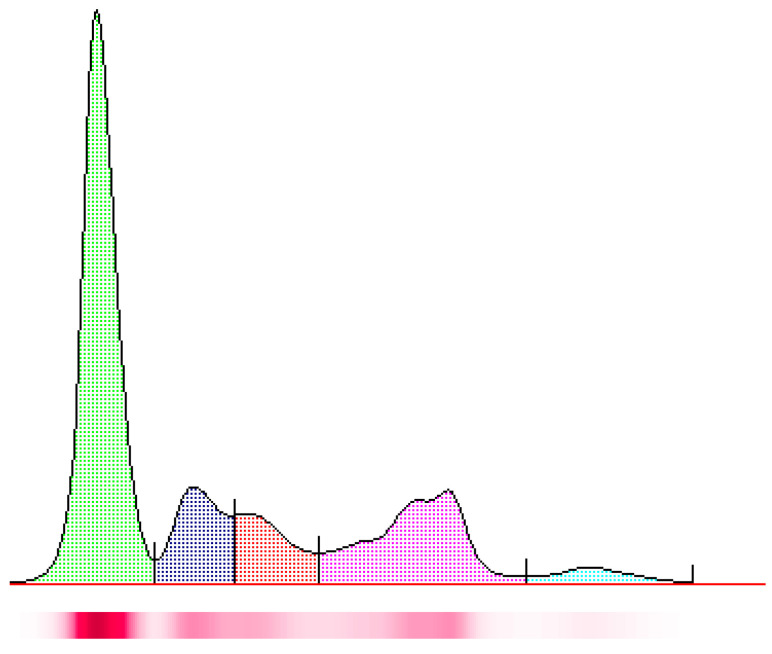
Electrophoretic pattern of a healthy individual of the species *Erinaceus europaeus*, respectively. Green—albumin; for globulins: dark blue—α1; red—α2; pink—β; light blue—γ.

In Figure 2, an example of the electrophoretic pattern of a healthy *E. europaeus* individual is presented. Medium values for each category are expressed in Table 2.

### 3.3. Influence of Sex and Age

Based on the Shapiro–Wilk test, it was determined that the parameters followed a normal distribution (*p* > 0.05).

Considering the sex of the animals, only ALP revealed a statistically significant difference between groups, being higher in males. No other parameters revealed a statistically significant association with sex.

Considering the animals’ age, regarding hematology, statistically significant differences were observed for MCV, MCH (both higher in juveniles), and RDW (higher in adults). Regarding clinical biochemistry, a statistically significant difference was observed for TP that was higher in adults. No other parameters revealed a statistically significant association with the age.

The results described above are expressed in Table 3.

## 4. Discussion

The Western European hedgehog (*Erinaceus europaeus*) is an insectivorous mammal widely distributed across Europe. A high number of hedgehogs live around urban areas, close to humans, where they are exposed to contaminants and biological agents that may affect their health status [17]. Moreover, hedgehogs can work as bioindicators and host multiple zoonotic agents, making them relevant in terms of a One Health approach [18,19]. The determination of RIs for usual hematological and biochemical parameters in wild healthy animals is the first step to identify potentially ill individuals. This is an important approach because those parameters, when altered, may be related to the presence of toxic or zoonotic agents in the environment [29,30]. That is the case of heavy metals such as zinc (Zn) and cadmium (Cd) that affect the liver and kidneys and can be detected by elevated levels of the biochemical parameters ALT and creatinine, respectively [31]. Identifying alterations in hedgehogs will allow the identification of other animal populations at risk of suffering the same type of exposure. In this way, the importance of the present study becomes clear in the context of One Health [24].

Existing works on RIs for hematology in this species are scarce. To the best of our knowledge, our study reports RIs for a wide range of parameters in comparison with previous investigations [17,27] and, to the best authors’ knowledge, is the first to calculate RIs for RDW, MPV, and PCT in *E. europaeus*.

In our study, considering age, statistically significant differences were found in MCV, MCH, and RDW. In MCV and MCH, the mean value was higher in juveniles than in adults and, in RDW, it was higher in adults compared with juveniles. In the study by Lewis et al. (2002) [27], a statistically significant difference was found only for HCT. The fact that the present study was carried out 2 decades after that of Lewis et al. [27], and used different instruments, may help to justify the differences found. However, the authors cannot rule out the possibility that environmental changes, exposure to biological or toxic agents, as well as access to new food sources due to repeated contact with humans may also explain the differences found [18,19]. Another justification for the differences found has to do with the genetic variability that occurs in animals of the same species in different geographic locations, as recently confirmed in a study carried out in different regions of the Iberian Peninsula [32].

The study by Rossi et al. (2014) [17] only included juvenile animals, so it was not possible to make this type of comparison.

Considering sex, in our work, there were no statistically significant differences for the analyzed parameters. The same observation was described by Lewis et al. (2002) [27]. However, Rossi et al. (2014) [17] obtained a difference for MCV, but it was considered not biologically relevant, and justified as a possible bias, a consequence of sex overlapping in their sample.

Studies describing RIs for biochemical parameters for *E. europaeus* are also scarce [17,26], as previous mentioned for hematology studies. The study by Rossi et al. (2014) [17] describes RI values for some biochemical parameters in this species. However, Rossi and collaborators analyzed fewer parameters when compared with our study. Additionally, Rossi and collaborators did not investigate the age effect (because only juveniles were included in their study), nor the sex effect, which is comprehensible given the fact that, using only juveniles, the animals did not reach sexual maturity. The study by Larsen and Tönder (1967) describes values of electrophoresis, but without RI determination.

To the best of our knowledge, our study is the first to describe RI values for AST, total bilirubin, sodium, potassium, and chloride for the species *E. europaeus*. It is important to note that this is the first time, in the literature, that information about the electrolyte panel is made available for this species.

Throughout our work, several important biochemical parameters were studied, providing valid information at the level of different organs and systems such as the liver and kidney, among others. Additionally, an investigation into the age and sex effect on the analyzed biochemical parameters was also performed. Considering age, statistically significant differences were found in TP, where the mean value was higher in adults compared with juveniles. TP refers to all proteins in plasma that are made up of albumin and globulins. Higher TP values in adults may be related to a greater number of globulins owing to a more developed immune system in adults compared with juveniles [33]. Although, in our study, we did not find a significant difference for globulin values between adults and juveniles, these values were in fact higher in adults, supporting this hypothesis.

Considering the sex effect, statistically significant differences were found in ALP, where the mean value was higher in males than in females. Typically, a higher ALP value is related to the presence of liver, bone, and other diseases [34]. However, there are no investigations in this species or others that indicate an increase or decrease in this parameter associated with sex. Therefore, we can conclude that the fact that this value is higher in males in our study is a coincidence related to the studied population and could be due to diet or some distinct environmental conditions [35].

Concerning protein electrophoresis, in a study in Bergen, Norway, by Larsen and Tönder (1967) [26], a paper protein electrophoresis analysis was conducted, in a population of 13 animals of the species *E. europaeus*. Contrary to our study, serum was used instead of plasma. Because of the differences in sample type and methodology, it was impossible for us to compare results. There are no more protein electrophoresis studies conducted in this species.

Although all animals included in this study were wildlife casualties, the samples used were collected only after a quarantine period, which would vary between 15 days and 3 months, depending on the cause of admittance. This time lapse between admittance and blood collection would give animals time to recover from illness, but also subject them to stress derived from captivity. Rasmussen et al. (2021) [36] showed that rehabilitated hedgehogs had higher levels of endogenous corticosterone when compared with wild-caught individuals. Although endogenous steroids can impact physical responses and lead to unbalances, the act of blood collection is considered a stressor and will contribute to this endogenous response [37]. Changes in the hematocrit and leucocyte count have been identified in a large set of species. This stress leukogram, however, varies from a transitory leukopenia, like in the rabbit, leukocytosis, as in camelids, or it can have no noticeable changes, as in rats [38]. It is also expectable to find increased blood levels of glucose and ALP with stress [39]. Isoflurane anesthesia can have an impact on measured blood parameters as it is known to increase serum glucose, AST, urea, nitrogen, and creatinine levels in rabbits [40] and to decrease erythrocyte parameters in ferrets [38].

Several of the results presented in both the Western European hedgehog and other species disagree with those observed in our work, supporting the existing differences between species and justifying the relevance of performing further studies. In addition, differences between methods and laboratories can also influence the results [41]. Moreover, the effects of stress in captivity and during handling on the hedgehogs investigated should be taken into consideration when interpreting the results, as it could influence the results and, thereby, the RIs determined [36].

It should be noted that the effects of sex and age on hematological and biochemical parameters are not necessarily uniform across geographic locations. So, for further studies, the inclusion of several different locations in an assessment or a meta-analysis should be noted. Therefore, it is really important that investigations are carried out in distinct countries/regions for a clear understanding of normal values, and thus cooperate for the improvement of the species’ conservative status.

## 5. Conclusions

The Western European hedgehog is a mammal with an extensive geographic distribution that, because of climatic changes and other aspects, began to live in urban areas near humans, where it is exposed to several anthropogenic pressures, suffering a progressive reduction in population. Hematological and biochemical RIs are scarce and there is a need to continue performing such types of investigations.

In this study, it was possible to establish reference intervals for a wide range of hematological and biochemical parameters covering the majority of the values used in the clinical practice. Moreover, we found out that the RIs obtained in this study do not always fit those previously published in the literature, for populations with different geographic locations, concluding the necessity to create appropriated RIs for the target population.

## Figures and Tables

**Table 1:** Reference intervals of hematological parameters in 37 healthy animals of the species *Erinaceus europaeus*.

Parameters	N	Mean ± SD	Median	Min–Max	RI	LRL 90% CI	URL 90% CI
RBC (M/μL)	37	7.10 ± 1.50	7.20	4.07–11.01	4.1–10.2	3.5–4.8	9.5–10.9
HCT (%)	37	30.20 ± 5.30	29.40	17.30–41.40	19.4–41.1	16.7–21.9	38.6–43.6
HGB (g/dL)	37	10.20 ± 1.80	10.00	5.70–13.90	6.6–13.8	5.7–7.5	13.0–14.6
MCV (fL)	37	42.90 ± 5.40	40.90	35.50–57.50	34.3–55.8	33.7–35.2	50.5–59.7
MCH (pg)	37	14.40 ± 1.70	14.00	11.70–19.20	12.0–19.1	11.8–12.3	17.4–20.9
MCHC (g/dL)	37	33.60 ± 1.30	33.60	31.40–36.70	30.9–36.4	30.2–31.6	35.8–37.0
RDW (%)	37	29.00 ± 3.20	28.90	24.20–37.80	22.5–35.6	21.0–24.1	34.0–37.1
%RETIC	37	3.60 ± 3.40	2.40	0.20–14.00	*	*	*
RETIC (K/μL)	37	222.70 ± 168.30	183.10	10.60–645.30	11.5–705.7	2.6–29.0	549.2–892.2
WBC (K/μL)	37	9.10 ± 3.20	8.60	2.22–15.31	2.5–15.7	1.0–4.0	14.2–17.3
%NEU	37	52.70 ± 14.10	54.50	5.00–74.20	23.8–81.6	16.1–30.6	74.4–88.6
%LYM	37	37.20 ± 11.00	36.20	19.30–60.10	14.6–59.9	9.6–20.0	54.5–65.1
%MONO	37	7.90 ± 6.20	6.70	2.80–42.30	3.5–24.4	3.1–4.2	14.3–44.9
%EOS	37	1.80 ± 1.80	1.00	0.00–6.50	2.4–26.9	2.0–3.0	14.7–75.2
%BASO	37	0.30 ± 0.40	0.20	0.00–1.00	*	*	*
NEU (K/μL)	37	4.90 ± 2.30	4.50	0.11–9.89	0.8–10	0.2–1.5	8.2–11.5
LYM (K/μL)	37	3.30 ± 1.40	3.30	1.15–6.28	0.4–6.3	0.0–0.9	5.5–6.8
MONO (K/μL)	37	0.60 ± 0.30	0.60	0.22–1.40	0.2–1.4	0.2–0.3	1.1–1.6
EOS (K/μL)	37	0.20 ± 0.20	0.10	0.00–0.48	*	*	*
BASO (K/μL)	37	0.00 ± 0.00	0.00	0.00–0.11	*	*	*
PLT (K/μL)	37	268.30 ± 129.90	237.00	6.00–620.00	31.3–567.1	3.2–71.5	472.2–660.3
MPV (fL)	33	15.60 ± 1.10	15.50	12.40–17.80	13.2–17.9	12.7–13.9	17.4–18.4
PCT (%)	33	0.40 ± 0.20	0.40	0.05–0.91	0.1–0.8	0.0–0.2	0.7–0.9

SD, standard deviation; Min, minimum; Max, maximum; LRL, lower reference limit; URL, upper reference limit; CI, confidence interval. * Non-computable. RBC, red blood cells; HCT, hematocrit; HGB, hemoglobin; MCV, mean cell volume; MCH, mean corpuscular hemoglobin; MCHC, mean corpuscular hemoglobin concentration; RDW, red blood cell distribution width; %RETIC, reticulocyte percent; RETIC, reticulocyte count; WBC, white blood cell; %NEU, neutrophil percent; %LYM, lymphocyte percent; %MONO, monocyte percent; %EOS, eosinophil percent; %BASO, basophil percent; NEU, neutrophil count; LYM, lymphocyte count; MONO, monocyte count; EOS, eosinophil count; BASO, basophil count; PLT, platelet count; MPV, mean platelet volume; PCT, plateletcrit.

**Table 2:** Reference intervals of biochemical parameters in 37 healthy animals of the species *Erinaceus europaeus*.

Parameters	N	Mean ± SD	Median	Min–Max	RI	LRL 90% CI	URL 90% CI
Glucose (mg/dL)	21	108.50 ± 24.10	109.00	44.20–141.91	57.0–160.0	43.0–72.2	144.0–175.3
TP (g/dL)	34	5.80 ± 1.20	5.90	3.55–8.59	3.4–8.3	2.8–3.9	7.6–8.9
Albumin (g/dL)	33	3.30 ± 0.60	3.20	2.29–4.46	2.1–4.5	1.8–2.4	4.2–4.8
ALT (U/L)	33	129.20 ± 61.20	125.00	36.40–298.00	*	*	*
ALP (U/L)	34	74.80 ± 48.00	59.50	18.00–203.10	19.2–217.8	17.8–22.7	166.3–281.4
Creatinine (mg/dL)	33	0.40 ± 0.20	0.30	0.10–0.77	0.1–0.9	0.1–0.1	0.7–1.0
Urea (mg/dL)	23	76.50 ± 42.70	68.00	16.20–215.40	*	*	*
Phosphorus (mg/dL)	33	7.70 ± 2.40	7.40	4.29–15.01	2.7–12.7	1.6–3.9	11.4–14.0
T. Calcium (mg/dL)	33	9.40 ± 1.60	9.90	2.55–11.35	5.6–11.5	1.4–7.4	11.0–11.7
Cholesterol (mg/dL)	15	134.90 ± 50.00	132.00	24.00–224.00	*	*	*
TG (mg/dL)	14	55.70 ± 14.70	56.00	18.00–79.00	22.9–88.5	10.6–35.7	75.1–100.9
Gamma-GT (U/L)	18	19.80 ± 17.90	8.70	2.60–56.00	1.8–151.8	*	*
Globulins (g/dL)	23	2.30 ± 1.00	2.10	0.00–3.97	0.4–4.7	0.1–0.9	3.6–5.9
AST (U/L)	9	19.60 ± 5.90	19.00	13.90–30.70	10.8–38.7	9.6–13.4	27.0–53.0
T. Bilirubin (mg/dL)	10	0.00 ± 0.00	0.00	0.01–0.03	*	*	*
Sodium (mmol/L)	20	142.90 ± 5.50	142.70	127.00–152.00	129.8–153.1	124.1–135.1	150.3–155.6
Potassium (mmol/L)	20	3.90 ± 0.70	3.80	2.15–5.44	2.5–5.4	2.0–2.9	4.9–5.9
Chloride (mmol/L)	20	110.20 ± 4.60	109.40	104.00–123.40	100.3–120.1	97.2–103.6	117.0–123.2
Protein electrophoresis in 12 cases							
Albumin (g/dL)	12	2.60 ± 0.40	2.50	1.91–3.14	1.7–3.5	1.4–2.1	3.1–3.8
α1-Globulin (g/dL)	12	0.60 ± 0.10	0.60	0.42–0.94	0.3–0.9	0.2–0.4	0.8–1.1
α2-Globulin (g/dL)	12	0.60 ± 0.10	0.60	0.32–0.76	0.2–0.9	0.1–0.4	0.8–1.0
β-Globulin (g/dL)	12	1.20 ± 0.50	1.10	0.70–2.37	0.6–3.4	0.5–0.8	1.8–8.9
γ-Globulin (g/dL)	12	0.20 ± 0.10	0.10	0.03–0.49	0.0–0.1	0.0–0.0	0.4–2.5

SD, standard deviation; Min, minimum; Max, maximum; LRL, lower reference limit; URL, upper reference limit; CI, confidence interval. * Non-computable. TP, total proteins; ALT, alanine aminotransferase; ALP, alkaline phosphatase; T. Calcium, total calcium; TG, triglycerides; AST, aspartate aminotransferase; T. Bilirubin, total bilirubin.

**Table 3:** Influence of sex and age on hematological and biochemical parameters in the species *Erinaceus europaeus*.

Category	Parameters	Mean ± SD	Mean ± SD	*p*
Hematology				
Age		Juveniles	Adults	
	MCV	44.95 ± 5.79	39.65 ± 3.88	0.007
	MCH	15.02 ± 1.72	13.49 ± 1.30	0.010
	RDW	27.99 ± 2.79	30.63 ± 3.41	0.021
Clinical Biochemistry				
Sex		Females	Males	
	ALP	58.50 ± 36.86	101.79 ± 55.84	0.012
Age		Juveniles	Adults	
	TP	5.47 ± 1.11	6.51 ± 1.34	0.034

## Data Availability

The data that support the findings of this retrospective study are available upon reasonable request to the authors.

## References

[1] Morris P. (2018). Hedgehog.

[2] Smith A.J. (1999). Husbandry and Nutrition of Hedgehogs. Veter Clin. N. Am. Exot. Anim. Pract..

[3] Paupério J., Vale-Gonçalves H.M., Cabral J.A., Mira A., Bencatel J., Bencatel J., Álvares F., Moura A.E., Barbosa A.M. (2017). Insetívoros. Atlas de Mamíferos de Portugal.

[4] Seddon J.M., Santucci F., Reeve N.J., Hewitt G.M. (2001). DNA footprints of *European hedgehogs*, *Erinaceus europaeus* and *E. concolor*: Pleistocene refugia, postglacial expansion and colonization routes. Mol. Ecol..

[5] Cabral M. (2005). Livro Vermelho dos Vertebrados de Portugal.

[6] Temple H.J., Cuttelod A. (2009). The Status and Distribution of Mediterranean Mammals.

[7] Hernández M.C., López P., Martin J., Barja I. (2020). Erizo común—*Erinaceus europaeus* Linnaeus, 1758. Enciclopedia Virtual de los Vertebrados Españoles.

[8] Pfäffle M. (2015). Influence of Parasites on Fitness Parameters of the European Hedgehog (Erinaceus europaeus).

[9] Young R.P., Davison J., Trewby I.D., Wilson G.J., Delahay R.J., Doncaster C.P. (2006). Abundance of hedgehogs (*Erinaceus europaeus*) in relation to the density and distribution of badgers (*Meles meles*). J. Zool..

[10] Rasmussen S.L., Berg T.B., Martens H.J., Jones O.R. (2023). Anyone Can Get Old—All You Have to Do Is Live Long Enough: Understanding Mortality and Life Expectancy in European Hedgehogs (*Erinaceus europaeus*). Animals.

[11] Rasmussen S.L., Berg T.B., Dabelsteen T., Jones O.R. (2019). The ecology of suburban juvenile European hedgehogs (*Erinaceus europaeus*) in Denmark. Ecol. Evol..

[12] Rasmussen S.L., Hallig J., van Wijk R.E., Petersen H.H. (2021). An investigation of endoparasites and the determinants of parasite infection in European hedgehogs (*Erinaceus europaeus*) from Denmark. Int. J. Parasitol. Parasites Wildl..

[13] Mariacher A., Santini A., Del Lesto I., Tonon S., Cardini E., Barone A., Eleni C., Fichi G., Perrucci S. (2021). Endoparasite Infections of the European Hedgehog (*Erinaceus europaeus*) in Central Italy. Animals.

[14] Hubert P., Julliard R., Biagianti S., Poulle M.-L. (2011). Ecological factors driving the higher hedgehog (*Erinaceus europeaus*) density in an urban area compared to the adjacent rural area. Landsc. Urban Plan..

[15] Poel J.L., Dekker J., Langevelde F. (2015). Dutch hedgehogs *Erinaceus europaeus* are nowadays mainly found in urban areas, possibly due to the negative Effects of badgers *Meles meles*. Wildl. Biol..

[16] Gazzard A., Yarnell R.W., Baker P.J. (2022). Fine-scale habitat selection of a small mammalian urban adapter: The West European hedgehog (*Erinaceus europaeus*). Mamm. Biol..

[17] Rossi G., Mangiagalli G., Paracchini G., Paltrinieri S. (2014). Hematologic and biochemical variables of hedgehogs (*Erinaceus europaeus*) after overwintering in rehabilitation centers. Veter Clin. Pathol..

[18] Delogu M., Cotti C., Lelli D., Sozzi E., Trogu T., Lavazza A., Garuti G., Castrucci M.R., Vaccari G., De Marco M.A. (2020). Eco-Virological Preliminary Study of Potentially Emerging Pathogens in Hedgehogs (*Erinaceus europaeus*) Recovered at a Wildlife Treatment and Rehabilitation Center in Northern Italy. Animals.

[19] Baptista C.J., Seixas F., Gonzalo-Orden J., Oliveira P. (2021). Can the European Hedgehog (*Erinaceus europaeus*) Be a Sentinel for One Health Concerns?. Biologics.

[20] Rasmussen S.L., Larsen J., Van Wijk R.E., Jones O.R., Berg T.B., Angen O., Larsen A.R. (2019). European hedgehogs (*Erinaceus europaeus*) as a natural reservoir of methicillin-resistant Staphylococcus aureus carrying mecC in Denmark. PLoS ONE.

[21] Larsen J., Raisen C.L., Ba X., Sadgrove N.J., Padilla-González G.F., Simmonds M.S.J., Loncaric I., Kerschner H., Apfalter P., Hartl R. (2022). Emergence of methicillin resistance predates the clinical use of antibiotics. Nature.

[22] Ruszkowski J., Hetman M., Turlewicz-Podbielska H., Pomorska-Mól M. (2021). Hedgehogs as a Potential Source of Zoonotic Pathogens—A Review and an Update of Knowledge. Animals.

[23] Baptista C.J., Oliveira P.A., Gonzalo-Orden J.M., Seixas F. (2023). Do Urban Hedgehogs (*Erinaceus europaeus*) Represent a Relevant Source of Zoonotic Diseases?. Pathogens.

[24] Horn P.S., Pesce A.J. (2003). Reference intervals: An update. Clin. Chim. Acta.

[25] Friedrichs K.R., Harr K.E., Freeman K.P., Szladovits B., Walton R.M., Barnhart K.F., Blanco-Chavez J. (2012). ASVCP reference interval guidelines: Determination of de novo reference intervals in veterinary species and other related topics. Veter Clin. Pathol..

[26] Larsen B., Tonder O. (1967). Serum Proteins in the Hedgehog. Acta Physiol. Scand..

[27] Lewis J.C.M., Norcott M.R., Frost L.M., Cusdin P. (2002). Normal haematological values of European hedgehogs (*Erinaceus europaeus*) from an English rehabilitation centre. Veter Rec..

[28] Haigh A., Kelly M., Butler F., O’Riordan R.M. (2014). Non-invasive methods of separating hedgehog (*Erinaceus europaeus*) age classes and an investigation into the age structure of road kill. Acta Thériol..

[29] Dowding C.V., Shore R.F., Worgan A., Baker P.J., Harris S. (2010). Accumulation of anticoagulant rodenticides in a non-target insectivore, the European hedgehog (*Erinaceus europaeus*). Environ. Pollut..

[30] Vermeulen F., Covaci A., D’Havé H., Brink N.W.V.D., Blust R., De Coen W., Bervoets L. (2010). Accumulation of background levels of persistent organochlorine and organobromine pollutants through the soil–earthworm–hedgehog food chain. Environ. Int..

[31] Al-Rikabi Z.G.K., Al-Saffar M.A., Abbas A.H. (2021). The Accumulative Effect of Heavy Metals on Liver and Kidney Functions. Med. Leg. Update.

[32] Araguas R.-M., Vidal O., García S., Sanz N. (2022). Genetic diversity and population structure of the Western European hedgehog, *Erinaceus europaeus*: Conservation status of populations in the Iberian Peninsula. Mamm. Biol..

[33] Garner B.C., Wiedmeyer C. (2011). Globulins. Clinical Veterinary Advisor.

[34] Sharma U., Pal D., Prasad R. (2014). Alkaline phosphatase: An overview. Indian J. Clin. Biochem..

[35] Ness R.D. (1999). Clinical Pathology and Sample Collection of Exotic Small Mammals. Veter Clin. N. Am. Exot. Anim. Pract..

[36] Rasmussen S.L., Kalliokoski O., Dabelsteen T., Abelson K. (2021). An exploratory investigation of glucocorticoids, personality and survival rates in wild and rehabilitated hedgehogs (*Erinaceus europaeus*) in Denmark. BMC Ecol. Evol..

[37] Romero L.M., Reed J.M. (2005). Collecting baseline corticosterone samples in the field: Is under 3 min good enough?. Comp. Biochem. Physiol. Part A Mol. Integr. Physiol..

[38] Campbell T.W., Thrall M.A., Weiser G., Allison R.W., Campbell T.W. (2022). Mammalian Hematology: Laboratory Animals and Miscellaneous Species. Section III: Hematology of Common Nondomestic Mammals, Birds, Reptiles, Fish, and Amphibians. Veterinary Hematology and Clinical Chemistry.

[39] Allison R.W., Thrall M.A., Weiser G., Allison R.W., Campbell T.W. (2022). Laboratory Evaluation of the Liver. Section V: Clinical Chemistry of Common Nondomestic Mammals, Birds, Reptiles, Fish, and Amphibians. Veterinary Hematology and Clinical Chemistry.

[40] Siegel A., Walton R.M. (2020). Hematology and Biochemistry of small mammals. Ferrets, Rabbits, and Rodents.

[41] Ferreira A.F., Queiroga F.L., Mota R.A., Rêgo E.W., Mota S.M., Teixeira M.G., Colaço A. (2018). Hematological profile of captive bearded capuchin monkeys (*Sapajus libidinosus*) from Northeastern Brazil. Cienc. Rural.

